# Novel Investigation of Influence of Torsional Load on Unbalance Fault Indicators for Induction Motors

**DOI:** 10.3390/s25072084

**Published:** 2025-03-26

**Authors:** Amir R. Askari, Len Gelman, Andrew D. Ball

**Affiliations:** 1 Department of Engineering, School of Computing and Engineering, The University of Huddersfield, Queensgate, Huddersfield HD1 3DH, UK; a.askari@hud.ac.uk (A.R.A.); a.ball@hud.ac.uk (A.D.B.); 2 Department of Mechanical Engineering, Hakim Sabzevari University, Sabzevar 9617976487, Iran

**Keywords:** fault diagnosis, induction motors, signal processing

## Abstract

This paper investigates how the torsional load affects two vibration-based unbalance fault indicators. This investigation is important for unbalance fault diagnosis in multiple constant load conditions, which are unavoidable for many rotating machines. Coupled flexural–torsional dynamics of an unbalanced disc–shaft system, as the representative of an induction motor, is investigated via a continuous shaft–beam model. Numerical investigations reveal that the fundamental rotating intensity of the transversal acceleration is independent of the torsional load. So, the novel speed-invariant version of this indicator, which is obtained by normalizing the fundamental rotating intensity by the fourth power of the rotational speed, is also load-independent. The comprehensive experimental trials confirm load-independency of the considered two unbalance fault indicators. The important novel outcome is that, by conducting numerical analysis and comprehensive experimental trials with a belt conveyor system under various constant loading conditions, the load-independency of the fundamental rotating harmonic intensity as well as novel speed-invariant unbalance feature are justified.

## 1. Introduction

All rotating machines could face a mass unbalance [1] that should be diagnosed and corrected. Otherwise, the level of stress in system components will be increased leading to a fatigue damage and failures [2].

Basically, given the 1X excitation coming from the mass unbalance centrifugal force, this fault is diagnosed by monitoring the vibration fundamental harmonic intensity [3,4,5,6,7,8]. Investigating an induction motor, Ewert [9] showed that aside from the spectrum analysis, the vibration bispectrum also contains the fault signature, and so, it can also be utilized for diagnosis. It is demonstrated that the new technique benefits from the ability to deal with signals with short durations and is more sensitive to the fault signature. This study is performed in stationary conditions, and the results are discussed for an unloaded motor.

Rafaq et al. [10] monitored a permanent magnet synchronous motor vibration signal. Investigating the machine vibration signal at different constant rotational speeds, the speed-dependency of the vibration fundamental harmonic intensity is referred to as the main signature distinguishing the mass unbalance fault from other load-related faults. This matches with the theory where the centrifugal excitation is proportional to the rotational speed in power two [11]. This study is performed in stationary conditions, and the results are reported for the two cases of half- and full-load conditions. Although the figures showed no considerable effect of load on the fundamental harmonic intensity, the influence of loading on the mass unbalance diagnostic feature is not addressed in this study.

Puerto-Santana et al. [12] also studied a general rotor system containing disc, shaft, and bearing elements operating at multiple constant rotational speeds. This study is also devoted to the stationary conditions where the diagnosis system was calibrated at different levels of the rotational speeds. Without investigating the influence of the torsional load, this study indicates that the unbalance monitoring must be performed in no-load conditions.

Aside from the works reviewed above, which performed in stationary conditions, Ewert et al. [13] investigated the unbalance fault in a dual rotor system in non-stationary conditions. They adopted short-time Fourier transform to process the vibration signal and diagnose the fault via monitoring the intensity of the fundamental harmonic. This study was carried out at no-active load conditions.

Li et al. [14] utilized G.H. Bladed 4.2 to develop a numerical model for wind turbines operating at variable speed conditions. They adopted the numerical model to obtain rotational inertia, rotational speed, and generator output power signals and then numerically re-constructed the aerodynamic torque signal. Using the order tracking method, they monitored the ratio of its first to third harmonic intensities to diagnose the unbalance fault. They reported that the higher unbalance severity, the higher the value of their diagnostic feature. Without a discussion on the influence of torsional load, this study diagnosed the unbalance fault when the turbine shaft was connected to the generator.

In general, having the speed signal either from a tachometer sensor [15,16] or extracted from the main signal [17,18,19,20,21], the order tracking method re-constructs the main signal in the order domain. Adopting a tacholess order tracking method, Wu et al. [22] extracted the intensity of the vibration fundamental harmonic associated with a motor-disc-bearing system. Compared to the short-time Fourier transform, they showed that their new technique can extract the feature much more accurately. However, the influence of the torsional load is not addressed in this study.

One of the main issues associated with unbalance fault diagnosis in variable speed machines is the speed-dependency of the vibration fundamental harmonic as the traditional fault indicator. Because, aside from the change in the fault severity, changing the rotational speed can change this diagnostic feature as well. Therefore, it will be difficult to judge the health of the machine via monitoring this fault indicator. To solve this problem, Askari et al. [23] proposed a novel speed-invariant diagnostic feature, whose magnitude is independent of the speed. Conducting comprehensive experimental trials, they showed their new feature behaves stably over the whole operational range of variable-speed large turbines. However, since the turbine is all the time connected to the generator and so under loading, the influence of the load has not been addressed in this work as well.

One of the most important research questions regarding vibration-based unbalance fault diagnosis is how the torsional load affects fault indicators. It is important to answer a basilar question: Is unbalance diagnostic features changed in multiple constant load conditions or not? To date, there exist very limited research efforts trying to answer this question. Salah et al. [24] investigated the influence of the torsional load on the unbalance fault indicator in a three-phase induction motor. Applying the load via a magnetic brake system, they showed that the vibration fundamental harmonic is independent of the torsional load. They claimed that this experimental investigation agrees with the theory where they modeled the motor as a simple mass–spring–damper system. However, regarding the simplicity of the theoretical background, where no coupled flexural–torsional vibrations are investigated, it is not possible to rely on the conclusion and claim that the level of vibration fundamental harmonic intensity is independent from the torsional load.

Suri [25] also experimentally investigated the influence of the torsional load on mechanical unbalance, eccentricity, and rolling element-bearing faults of an induction motor. This study reported that despite the current signal, no clear effect on the vibrational signal is observed.

In summary, to the best of the authors’ knowledge, the influence of the torsional load on the vibration fundamental harmonic intensity has not been theoretically and experimentally addressed. Therefore, this study aims to address this influence for the first time in a wide range through theory and comprehensive experimental trials. Firstly, the coupled flexural–torsional dynamics of an unbalanced disc–shaft system under torsional load are theoretically investigated. The governing equations of motion are obtained and numerically solved via the Galerkin method. Taking the Fourier transform from the solutions, the influence of the torsional load on the intensity of the fundamental harmonic is investigated. The theory is validated by experimental observations: comprehensive experimental trials are conducted on conveyor belt systems under different levels of loading. Referring to the influence of loading on reducing the system rotational speed, the experimental investigations are performed through both the traditional and the novel unbalance diagnostic features [23]. Given the unavoidable speed fluctuations during real operational conditions, the collected vibration data from the belt conveyor system are processed via the short-time chirp Fourier transform technique, which was proposed by L. Gelman et al. [26] and benefits from accurate extraction of the harmonic intensities.

The novelties of the paper are as follows:

- A coupled flexural–torsional dynamical model for an unbalanced disc–shaft system as the representative for an unbalanced induction motor.
- Investigation of influence of the torsional load on unbalance fault diagnosis indicators, the conventional 1X magnitudes, and the speed-invariant feature [23] for the first time, wideranging by theory and comprehensive experimental trials.
- Comparison of the influence of the torsional load on the vibration fundamental rotating harmonic intensity and the speed-invariant unbalance diagnostic feature [23].

Given the aforementioned novelties, the objectives of the present work are

- To derive the coupled flexural–torsional governing equations of motion corresponding to an unbalanced disc–shaft system under torsional load using the Hamilton principle.
- To provide numerical solution for the governing equations of motion using Galerkin decomposition method.
- To numerically investigate the influence of the torsional load on the intensity of the vibration fundamental harmonic.
- To numerically investigate the influence of the torsional load on the novel speed-invariant unbalance diagnostic feature [23].
- To collect vibration data from a belt conveyor system under different torsional loads.
- To process the vibration data collected from the belt conveyor system.
- To subtract the average local interference level around the fundamental peak and obtain the feature values.
- To normalize the net feature values by the average surrounding interference level.
- To evaluate the Fisher criterion [27] as well as the separation probability [28] between the feature histograms as measures justifying how far two features are separated from each other.
- To experimentally investigate the influence of the applied load on the conventional unbalance diagnostic feature.
- To experimentally investigate the torsional load effect on the novel speed-invariant unbalance diagnostic feature [23].

The rest of the paper is organized as follows: In Section 2, the theoretical model is proposed. This section also deals with the numerical solution to the derived partial differential equations. Section 3 describes the vibration data capturing system installed over the belt conveyor. Numerical results and experimental observations are discussed in Section 4. The main conclusions are summarized in Section 5.

## 2. Theoretical Model

Induction motors can be considered the most usual parts in rotating machines. This machine includes a rotor assembly driven by a magnetic torque. So, an unbalanced disc–shaft system is considered the representative of an unbalanced induction motor. This study does not account for other rotating machines including those containing gears.

**Figure 1:**
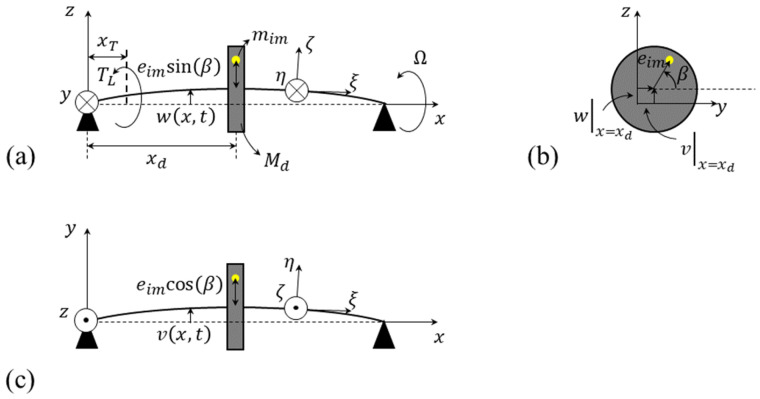
Schematic of the disc–shaft system in its deformed configuration: (**a**) the front view, (**b**) the side view, and (**c**) the top view.

Figure 1 illustrates a schematic of an unbalanced disc–shaft system. To obtain the coupled flexural–torsional governing equations of motion, a space-fixed x−y−z coordinate system is attached to the left-hand side of the shaft. In addition, a body-fixed ξ−η−ζ is attached to the center of the shaft cross-section. Let $u\left(x,t\right),v\left(x,t\right),\;and\;w\left(x,t\right)$ be the displacement components of a point located on the shaft centerline along the *x*, *y*, and *z* directions, respectively, and $\varphi \left(x,t\right)$ describes the shaft torsional deformation. So, according to the Euler–Bernoulli beam theory, where the shaft slopes in x−y and x−z planes are equal to $\psi \left(x,t\right)=\frac{\partial v\left(x,t\right)}{\partial x}$ and $\theta \left(x,t\right)=-\frac{\partial w\left(x,t\right)}{\partial x}$, respectively, the components of the displacement field, $u_{1}\left(x,t\right)$, $u_{2}\left(x,t\right)$, and $u_{3}\left(x,t\right)$, which are associated with an arbitrary point located on the shaft cross-section along the *x*, *y*, and *z* directions, respectively, take the form of [29]

(1) $$u_{1}\left(x,t\right)=u\left(x,t\right)-y\frac{\partial v\left(x,t\right)}{\partial x}-z\frac{\partial w\left(x,t\right)}{\partial x},$$

(2) $$u_{2}\left(x,t\right)=v\left(x,t\right)-z\varphi \left(x,t\right),$$

(3) $$u_{3}\left(x,t\right)=w\left(x,t\right)+y\varphi \left(x,t\right).$$

Considering $\left(\psi ,\theta ,\beta \right)$ as the well-known triple set of Euler’s angles [11], the angle $\beta \left(x,t\right)$, describing the torsional deformation, i.e., $\varphi \left(x,t\right)$, superimposed over the rigid-body rotation around the shaft centerline, i.e., Ω, takes the form of

(4) $$\beta \left(x,t\right)=\Omega t+\varphi \left(x,t\right).$$

Having the displacement field components, the disc–shaft system kinetic energy expression, *K*, yields [30]

(5) $$\begin{array}{ll} K=\frac{1}{2}\rho \int_{0}^{L} & \left\{A\left[{{\left(\frac{\partial u}{\partial t}\right)}^{2}+\left(\frac{\partial v}{\partial t}\right)}^{2}+{\left(\frac{\partial w}{\partial t}\right)}^{2}\right]+J_{sh}\omega_{1}^{2}+I_{sh}\left(\omega_{2}^{2}+\omega_{3}^{2}\right)\right\}dx\\ & +\frac{1}{2}M_{d}\left[{{\left({\left.\frac{\partial u}{\partial t}\right|}_{x=x_{d}}\right)}^{2}+\left({\left.\frac{\partial v}{\partial t}\right|}_{x=x_{d}}\right)}^{2}+{\left({\left.\frac{\partial w}{\partial t}\right|}_{x=x_{d}}\right)}^{2}\right]\\ & +\frac{1}{2}J_{d}{\left({\left.\omega_{1}\right|}_{x=x_{d}}\right)}^{2}+\frac{1}{2}I_{d}\left[{\left({\left.\omega_{2}\right|}_{x=x_{d}}\right)}^{2}+{\left({\left.\omega_{3}\right|}_{x=x_{d}}\right)}^{2}\right]\\ & +\frac{1}{2}m_{im}\left[{{\left(\frac{\partial }{\partial t}\left({\left.u\right|}_{x=x_{d}}\right)\right)}^{2}+\left(\frac{\partial }{\partial t}\left({\left.v\right|}_{x=x_{d}}+e_{im}cos\left({\left.\beta \right|}_{x=x_{d}}\right)\right)\right)}^{2}+{\left(\frac{\partial }{\partial t}\left({\left.w\right|}_{x=x_{d}}+e_{im}sin\left({\left.\beta \right|}_{x=x_{d}}\right)\right)\right)}^{2}\right], \end{array}$$

in which *L* denotes the shaft length, $x_{d}$ refers to the location of the disc, ρ is the shaft density, and A is its cross-sectional area, which equals $\pi r^{2}$, where *r* is the shaft radius. $I_{sh}$ is the second moment of the shaft cross-sectional area around the transversal η or ζ axes, which equals $0.25\pi r^{4}$. $J_{sh}$ is also the polar second moment of the cross-sectional area, which equals $0.5\pi r^{4}$. $M_{d}$, $I_{d}$, and $J_{d}$ denote the disc mass, its second moment of inertia around the transversal η or ζ axes, which is equal to $0.25M_{d}r_{d}^{2}$, and its polar mass moment of inertia, which is equal to $0.5M_{d}r_{d}^{2}$, respectively. $m_{im}$ is the mass unbalance located at the distance of $e_{im}$ from the disc center. $\omega_{1}$, $\omega_{2}$, and $\omega_{3}$ are the angular velocity components in the body-fixed local coordinate frame ξ−η−ζ and are given by [31]

(6) $$\omega_{1}=\frac{\partial \beta }{\partial t}-\frac{\partial \psi }{\partial t}sin\left(\theta \right),$$

(7) $$\omega_{2}=\frac{\partial \psi }{\partial t}sin\left(\beta \right)cos\left(\theta \right)+\frac{\partial \theta }{\partial t}cos\left(\beta \right),$$

(8) $$\omega_{3}=\frac{\partial \psi }{\partial t}cos\left(\beta \right)cos\left(\theta \right)-\frac{\partial \theta }{\partial t}sin\left(\beta \right).$$

Given the displacement field in Equations (1)–(3), the non-zero strain components associated with the shaft deformation can be obtained as [32]

(9) $$\varepsilon_{x}=\frac{\partial u}{\partial x}-y\frac{\partial^{2}v}{\partial x^{2}}-z\frac{\partial^{2}w}{\partial x^{2}},$$

(10) $$\gamma_{xy}=-z\frac{\partial \varphi }{\partial x},$$

(11) $$\gamma_{xz}=y\frac{\partial \varphi }{\partial x}.$$

where $\varepsilon_{x}$ is the normal strain along the *x* direction, $\gamma_{xy}$ is the shear strain on y−z plan along the *y* direction, and $\gamma_{xz}$ denotes the shear component on the same plane along the *z* direction.

The stress components conjugated with the strains given in Equations (9)–(11) are given by [32]

(12) $$\sigma_{x}=E\varepsilon_{x},$$

(13) $$\tau_{xy}=G\gamma_{xy},$$

(14) $$\tau_{xz}=G\gamma_{xz}.$$

where *E* is the shaft elasticity modulus, and *G* denotes its shear modulus.

Taking the non-zero strain and stress components into account, the elastic strain energy of the shaft, *U*, is given by [32]

(15) $$U=\frac{1}{2}\int_{0}^{L}\left\{EA{\left(\frac{\partial u}{\partial x}\right)}^{2}+EI_{sh}\left[{\left(\frac{\partial^{2}v}{\partial x^{2}}\right)}^{2}+{\left(\frac{\partial^{2}w}{\partial x^{2}}\right)}^{2}\right]+GJ_{sh}{\left(\frac{\partial \varphi }{\partial x}\right)}^{2}\right\}dx.$$

The works conducted by external factors like damping forces and the torque load are given by

(16) $$W_{ext}=\int_{0}^{L}\left\{-C_{u}\frac{\partial u}{\partial t}u-C_{v}\frac{\partial v}{\partial t}v-C_{w}\frac{\partial w}{\partial t}w-C_{\varphi }\frac{\partial \varphi }{\partial t}\varphi \right\}dx+T_{L}{\left.\varphi \right|}_{x=x_{T}},$$

where $C_{u}$ is the axial damping coefficient. In addition, $C_{v}$ and $C_{w}$ are the transversal damping coefficients along the *y* and *z* directions, respectively. $C_{\varphi }$ also denotes the torsional damping coefficient. $T_{L}$ is the torsional load, and $x_{T}$ specifies the location on which this torque is applied.

According to the Hamilton principle [32], the dynamics of deformable objects within the time interval of $\left(t_{i},t_{f}\right)$ is governed by

(17) $$\int_{t_{i}}^{t_{f}}\delta \left(K-U+W_{ext}\right)dt=0,$$

where δ refers to the variation operator.

Substituting Equations (5), (15), and (16) into Equation (17), applying the fundamental lemma of the calculus of variation [32], approximating $sin\left(\varphi \right)≅\varphi$ and $cos\left(\varphi \right)≅1$, and following some straightforward mathematical manipulations, the governing equations of motion are given by

(18) $$\delta u:EAu^{\prime \prime }+C_{u}\dot{u}+\left[\rho A+\left(M_{d}+m_{im}\right)H\left(x-x_{d}\right)\right]\ddot{u}=0,$$

(19) $$\begin{array}{ll} \delta v:EI_{sh}v^{⁗}+C_{v}\dot{v} & +\left[\rho A+\left(M_{d}+m_{im}\right)H\left(x-x_{d}\right)\right]\ddot{v}\\ & -\left[\rho I_{sh}+I_{d}H\left(x-x_{d}\right)\right]\left({\ddot{v}}^{\prime \prime }+2\Omega {\dot{w}}^{\prime \prime }+2\ddot{\varphi }w^{\prime \prime }+2{\ddot{\varphi }}^{\prime }w^{\prime }+2{\dot{\varphi }}^{\prime }{\dot{w}}^{\prime }+2\dot{\varphi }{\dot{w}}^{\prime \prime }\right)\\ & =m_{im}e_{im}H\;(x-x_{d})\left[\left(\Omega^{2}+2\Omega \dot{\varphi }\right)cos\left(\Omega t\right)+\left(\ddot{\varphi }-\Omega^{2}\varphi \right)sin\left(\Omega t\right)\right], \end{array}$$

(20) $$\begin{array}{ll} \delta w:EI_{sh}w^{⁗} & +C_{w}\dot{w}+\left[\rho A+\left(M_{d}+m_{im}\right)H\left(x-x_{d}\right)\right]\ddot{w}\\ & -\left[\rho I_{sh}+I_{d}H\left(x-x_{d}\right)\right]\left({\ddot{w}}^{\prime \prime }-2\Omega {\dot{v}}^{\prime \prime }-2{\dot{\varphi }}^{\prime }{\dot{v}}^{\prime }-2\dot{\varphi }{\dot{v}}^{\prime \prime }\right)\\ & =m_{im}e_{im}H\;\left(x-x_{d}\right)\left[\left(\Omega^{2}+2\Omega \dot{\varphi }\right)sin\left(\Omega t\right)-\left(\ddot{\varphi }-\Omega^{2}\varphi \right)cos\left(\Omega t\right)\right], \end{array}$$

(21) $$\delta \varphi :\text{-}GJ_{sh}\varphi^{\prime \prime }+C_{\varphi }\dot{\varphi }+m_{im}e_{im}^{2}H\left(x-x_{d}\right)\ddot{\varphi }+\left[\rho J_{sh}+J_{d}H\left(x-x_{d}\right)\right]\;\left(\ddot{\varphi }+{\ddot{v}}^{\prime }w^{\prime }+\dot{v}\prime \dot{w}\prime \right)=T_{L}H\;(x-x_{T})+m_{im}e_{im}H\;\left(x-x_{d}\right)\left[\ddot{v}sin\left(\Omega t\right)-\ddot{w}cos\left(\Omega t\right)\right].$$

where the dot and prime superscripts refer to the partial differentiation with respect to *t* and *x*, respectively. Also, $H\left(x\right)$ is the Heaviside function. As it is seen, the axial deformation is decoupled from the transversal and torsional deformations.

Given the fact that the axial motion is not coupled with the transversal and torsional motions of the system, Equations (19)–(21) are only considered in this study. These equations are coupled and account for gyroscopic motion of the disc–shaft system.

All the rotors are supported by bearings, which are usually modeled by the simply supported boundary conditions [33]. So, the *i*th system eigen modes for both the transversal and torsional deformations take the sinusoidal form as $sin\left(\frac{i\pi x}{L}\right)$ [33]. Considering systems in which the rotational speed does not pass their lowest resonance frequency, like the operational situation of most of the induction AC gearmotors, the only dominant mode will be the first one [29,31]. That is, the transversal as well as the torsional mode shapes of the shaft are obtained as $sin\left(\frac{\pi x}{L}\right)$. So, one can write the following:

(22) $$v\left(x,t\right)≅sin\left(\frac{\pi x}{L}\right)v_{mid}\left(t\right),$$

(23) $$w\left(x,t\right)≅sin\left(\frac{\pi x}{L}\right)w_{mid}\left(t\right),$$

(24) $$\varphi \left(x,t\right)≅sin\left(\frac{\pi x}{L}\right)\varphi_{mid}\left(t\right).$$

where $v_{mid}$, $w_{mid}$, and $\varphi_{mid}$ are the unknown generalized coordinates, which should be obtained during the solution procedure.

It is notable that these generalized coordinates refer to the transversal deflection of the shaft mid-point along the *y* and *z* axe as well as the torsional deformation at the middle of the shaft, respectively.

Taking into account the aforementioned sinusoidal mode shapes as the admissible basis functions in the Galerkin method [32,34], the reduced equations of motion can be obtained as

(25) $$\begin{array}{ll} K_{y}v_{mid}+C_{y}{\dot{v}}_{mid} & +M_{y}{\ddot{v}}_{mid}+B_{1}{\dot{w}}_{mid}+B_{2}\left({\ddot{\varphi }}_{mid}w_{mid}+{\dot{\varphi }}_{mid}{\dot{w}}_{mid}\right)\\ & =m_{im}e_{im}\left[\left(\Omega^{2}+2\Omega sin\left(\frac{\pi x_{d}}{L}\right){\dot{\varphi }}_{mid}\right)cos\left(\Omega t\right)+\left({\ddot{\varphi }}_{mid}-\Omega^{2}\varphi_{mid}\right)sin\left(\frac{\pi x_{d}}{L}\right)sin\left(\Omega t\right)\right]sin\left(\frac{\pi x_{d}}{L}\right), \end{array}$$

(26) $$\begin{array}{ll} K_{z}w_{mid}+C_{z}{\dot{w}}_{mid} & +M_{z}{\ddot{w}}_{mid}-B_{1}{\dot{v}}_{mid}-B_{2}{\dot{\varphi }}_{mid}{\dot{v}}_{mid}\\ & =m_{im}e_{im}\left[\left(\Omega^{2}+2\Omega sin\left(\frac{\pi x_{d}}{L}\right){\dot{\varphi }}_{mid}\right)sin\left(\Omega t\right)-\left({\ddot{\varphi }}_{mid}-\Omega^{2}\varphi_{mid}\right)sin\left(\frac{\pi x_{d}}{L}\right)cos\left(\Omega t\right)\right]sin\left(\frac{\pi x_{d}}{L}\right), \end{array}$$

(27) $$\begin{array}{ll} K_{t}\varphi_{mid}+C_{t}{\dot{\varphi }}_{mid} & +M_{t}{\ddot{\varphi }}_{mid}+B_{3}\left({\ddot{v}}_{mid}w_{mid}+{\dot{v}}_{mid}{\dot{w}}_{mid}\right)\\ & =T_{L}sin\left(\frac{\pi x_{T}}{L}\right)\\ & +m_{im}e_{im}\;\left[{\ddot{v}}_{mid}sin\left(\Omega t\right)-{\ddot{w}}_{mid}cos\left(\Omega t\right)\right]{sin}^{2}\left(\frac{\pi x_{d}}{L}\right). \end{array}$$

in which

(28) $$K_{y}=EI_{sh}{\left(\frac{\pi }{L}\right)}^{4}\int_{0}^{L}{sin}^{2}\left(\frac{\pi x}{L}\right)dx,\;{C_{y}=C}_{v}\int_{0}^{L}{sin}^{2}\left(\frac{\pi x}{L}\right)dx,\;\;M_{y}=\rho A\int_{0}^{L}{sin}^{2}\left(\frac{\pi x}{L}\right)dx+\left(M_{d}+m_{im}\right){sin}^{2}\left(\frac{\pi x_{d}}{L}\right)+\rho I_{sh}{\left(\frac{\pi }{L}\right)}^{2}\int_{0}^{L}{sin}^{2}\left(\frac{\pi x}{L}\right)dx+{\left(\frac{\pi }{L}\right)}^{2}{sin}^{2}\left(\frac{\pi x_{d}}{L}\right)I_{d},\;\;B_{1}=2\Omega {\left(\frac{\pi }{L}\right)}^{2}\left[\rho I_{sh}\int_{0}^{L}{sin}^{2}\left(\frac{\pi x}{L}\right)dx+I_{d}{sin}^{2}\left(\frac{\pi x_{d}}{L}\right)\right],\;B_{2}=-2\rho I_{sh}{\left(\frac{\pi }{L}\right)}^{2}\int_{0}^{L}\left[{cos}^{2}\left(\frac{\pi x}{L}\right)sin\left(\frac{\pi x}{L}\right)-{sin}^{3}\left(\frac{\pi x}{L}\right)\right]dx-2{\left(\frac{\pi }{L}\right)}^{2}\left[{cos}^{2}\left(\frac{\pi x_{d}}{L}\right)sin\left(\frac{\pi x_{d}}{L}\right)-{sin}^{3}\left(\frac{\pi x_{d}}{L}\right)\right]I_{d},\;K_{z}=EI_{sh}{\left(\frac{\pi }{L}\right)}^{4}\int_{0}^{L}{sin}^{2}\left(\frac{\pi x}{L}\right)dx,\;{C_{z}=C}_{w}\int_{0}^{L}{sin}^{2}\left(\frac{\pi x}{L}\right)dx,\;\;M_{z}=\rho A\int_{0}^{L}{sin}^{2}\left(\frac{\pi x}{L}\right)dx+\left(M_{d}+m_{im}\right){sin}^{2}\left(\frac{\pi x_{d}}{L}\right)+\rho I_{sh}{\left(\frac{\pi }{L}\right)}^{2}\int_{0}^{L}{sin}^{2}\left(\frac{\pi x}{L}\right)dx+{\left(\frac{\pi }{L}\right)}^{2}{sin}^{2}\left(\frac{\pi x_{d}}{L}\right)I_{d},\;K_{t}={GJ_{sh}\left(\frac{\pi }{L}\right)}^{2}\int_{0}^{L}{sin}^{2}\left(\frac{\pi x}{L}\right)dx,C_{t}=C_{\varphi }\int_{0}^{L}{sin}^{2}\left(\frac{\pi x}{L}\right)dx,\;\;M_{t}=\rho J_{sh}\int_{0}^{L}{sin}^{2}\left(\frac{\pi x}{L}\right)dx+J_{d}{sin}^{2}\left(\frac{\pi x_{d}}{L}\right)+m_{im}e_{im}^{2}{sin}^{2}\left(\frac{\pi x_{d}}{L}\right),\;B_{3}=\rho {J_{sh}\left(\frac{\pi }{L}\right)}^{2}\int_{0}^{L}{cos}^{2}\left(\frac{\pi x}{L}\right)sin\left(\frac{\pi x}{L}\right)dx+{J_{d}cos}^{2}\left(\frac{\pi x_{d}}{L}\right)sin\left(\frac{\pi x_{d}}{L}\right).$$

The unbalance fault is reflected on the axes, placed within the plane perpendicular to the shaft direction, i.e., either the *y* or the *z* axis, as illustrated in Figure 1. Regarding the symmetry of the shaft cross-section, there is no difference between these two axes from a theoretical point of view [23]. However, in a practical situation, this fault is only reflected on the direction with lower support stiffness [23]. Therefore, just for the sake of brevity, this study only focuses on the *y* axis. Because, from a theoretical point of view, there is no difference between the *y* and *z* axes results.

Equations (25)–(27) govern the flexural–torsional dynamics of an unbalanced disc–shaft system as the representative of an unbalanced induction motor. Solution to this system of coupled nonlinear ordinary differential equations provides the possibility of investigating the influence of the torsional load on the intensity of the fundamental transversal vibration rotational harmonic, as the conventional unbalance fault indicator, and the novel speed-invariant unbalance diagnostic feature [23].

## 3. Experimental Analysis

### 3.1. Experimental Setup

To verify the theory, a gearmotor with a permissible level of unbalance that is running a belt conveyor system under different levels of additional loading is investigated.

**Figure 2:**
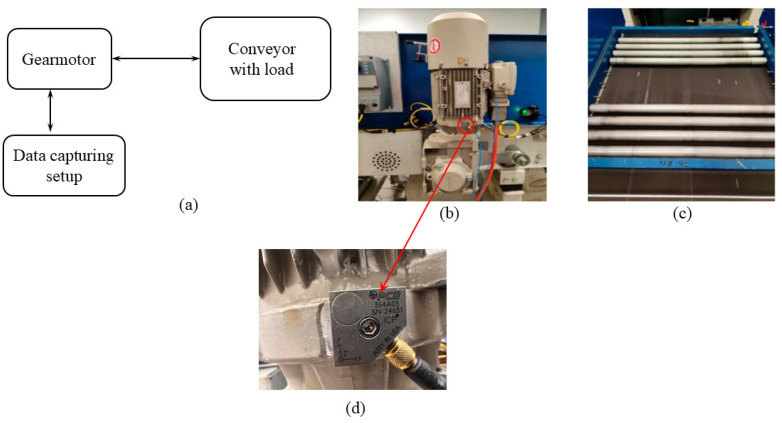
The experimental setup: (**a**) the principal diagram, (**b**) the gearmotor, (**c**) the loaded belt conveyor system, and (**d**) the triaxial accelerometer.

Figure 2 illustrates the experimental system setup. As it is seen from Figure 2a, the gearmotor, on which the triaxial accelerometer has been installed, runs the loaded conveyor. According to Figure 2b, the conveyor is driven by the Siemens-JKE2104 gearmotor, which has been sourced from the UK and contains a two-stage gearbox, the first stage gear ratio is 18/37 and the second one is 17/46, and a three-phase AC induction motor with nominal torque of 40.6 N.m and nominal speed of 1440 rpm. The first gearbox stage consists of an 18-teeth helical pinion, engaged with a 37-teeth helical gear (the helix angle is 30°). The second stage consists of a 17-teeth bevel pinion, engaged with a 46-teeth bevel gear. The rotor is supported by two single-row deep groove ball bearings. In addition, the first and the second stage shafts of the gearbox are placed on pairs of tapered rollers and single-row deep groove ball bearings, respectively.

Aside from the no-additional-load case, the experiments are performed under three additional loadings, consisting of 20 kg, 30 kg, and 50 kg on the conveyor. These are applied via a series of rollers surrounded by a frame placed on the belt conveyor (Figure 2c).

Applying different torsional loads to the gearmotor with a permissible level of unbalance, the transverse vibration data are collected and processed, and both the conventional and the speed-invariant unbalance fault indicators [23] are extracted. Comparing the fault indicators under different levels of additional loadings, the influence of torsional loading on the indicators will finally be addressed.

The gearmotor vibration data are collected via a triaxial piezoelectric accelerometer, bolted on the motor shell, to be as close as possible to the rotor. The *X* axis of the accelerometer is along the conveyor movement direction, *Y* axis is along the motor shaft, and *Z* axis is horizontal perpendicular to the conveyor movement direction (see Figure 2d). The accelerometer model is 354A05, PCB Piezotronics Inc. which has been sourced from the Stevenage, UK. The accelerometer specifications are detailed in Table 1.

**Figure 3:**
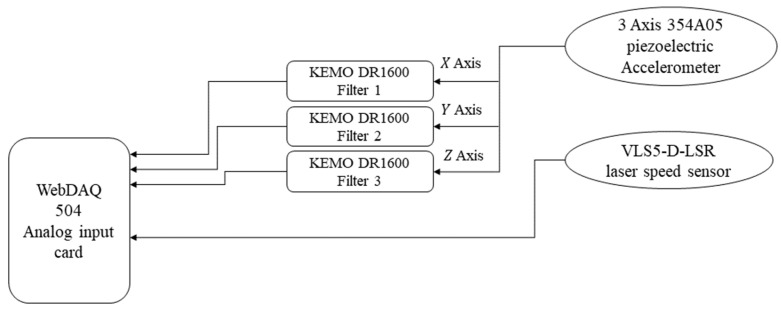
Schematic of the data capturing system.

Figure 3 illustrates the details of the data capturing system, including (i) the triaxial accelerometer, (ii) a speed sensor, (iii) KEMO filters, and (iv) a web data acquisition system. The conveyor shaft speed is measured via the VLS5-D-LSR laser speed sensor. The motor shaft speed is obtained using the gear ratios given above. That is,

(29) $$f=f_{c}\times \frac{37}{18}\times \frac{46}{17},$$

where *f* is the motor shaft speed in Hz (Ω=2πf), and $f_{c}$ denotes the conveyor driving roller speed, measured by laser speed sensor.

The speed sensor, which was available in house, measures the rotational speeds ranging from 3 to 250,000 rpm. However, its measurement ability is much more than required for the speed measurement in the present system. The optical range of the sensor covers the interval from 50 mm to 2000 mm, and it can be positioned at angles up to ±80° relative to the target, which allows flexible placement even in areas with difficult access. The sensor emits a high-visibility red laser beam.

The accelerometer outputs are amplified by three DR 1600 KEMO anti-aliasing filters with 3W power input. These filters, whose bandwidths are 500 kHz, are hired to delimitate the frequency bandwidth of the signals. The operating temperature range of these filters covers the interval from −10 °C to 45 °C. The total harmonic distortion of these filters is lower than 0.003%. The gains of these filters are set to ×20, and their cut-off frequencies are set to 8 kHz.

To sample the analog outputs of the vibration and speed sensors, a four-input WebDAQ 504 data acquisition card with sampling rate 51.2 kS/s is employed. This card, which is designed for remote monitoring purposes, offers 4 IEPE inputs. It benefits from simultaneous sampling of the four channels with 24-bit resolution. The range of the card input voltage is +/−5 V. Its input frequency range is 13 MHz.

### 3.2. Processing the Experimental Signals

Given the motor speed fluctuations, which are unavoidable in operating situations, a non-stationary processing tool is adopted here. Taking into account the inaccuracies involved in the short-time Fourier transform, as the non-stationary processing tool [22], the short-time chirp Fourier transform [26] is adopted in this study, which is effectively used for fault diagnosis under variable frequency conditions [35,36]. Let $q\left(t\right)$ be a digital time signal, and the short-time chirp Fourier transform is given by [26]

(30) $$Q\left(f,T\right)=\frac{1}{T}\int_{-\infty }^{+\infty }h\left(t-T\right)q\left(t\right)e^{-2\pi j\left(\int_{0}^{t}f\left(\tau \right)d\tau \right)}dt,$$

in which $j=\sqrt{-1}$, $h\left(t\right)$ denotes the time window with the duration of *T*, and *f* is the instantaneous frequency in Hz measured by a tachometer.

The net peak value corresponding to the fundamental harmonic is obtained by subtracting the average level of its surrounding interference, which is proposed by L. Gelman [23,37]:

(31) $$N_{ave}=\frac{\sum_{i=1}^{n}N_{i}^{left}+\sum_{i=1}^{n}N_{i}^{right}}{2n},$$

where $N_{i}^{left}$ and $N_{i}^{right}$ are the intensities of the *i*th component from the peak on its left- and right-hand sides, respectively.

Leaving the nearest component to 1X peak at each side, the value of *n* is set to 1 in this study. So, the net peak value yields [23,37,38]

(32) $$P_{net}=\sqrt{P^{2}-N_{ave}^{2}},$$

where *P* denotes the peak value.

After determining the net peak value, it is normalized by the average surrounding local interference level to obtain the conventional unbalance fault indicator [23]. Doing so, one obtains

(33) $$S=\frac{P_{net}}{N_{ave}},$$

Regarding the influence of load on reducing the rotational speed to obtain a speed-invariant unbalance fault indicator, in frames of the limitations mentioned in Ref. [23], the conventional indicator in Equation (33) should be divided by the average rotational speed to the power of four over each time window [23]. That is,

(34) $$S_{N}=\frac{S}{{\bar{f}}^{4}},$$

where $\bar{f}$ denotes the mean value of the rotational speed over each time window.

Since *S* is dimensionless, the unit of $S_{N}$, the speed-invariant unbalance fault indicator [23], is Hz^−4^ or s^4^.

## 4. Results and Discussions

The system of second-order ordinary differential equations in Equations (25)–(27) contains three nonlinear initial values, which can be numerically solved using the fourth-order Runge–Kutta method [39,40]. Assuming zero initial conditions, this system of equations is re-written as a system of six first-order equations and then solved via MATLAB, version R2024a, command ODE45. Adopting the solutions together with Equations (25)–(27), the accelerations are determined. Taking the Fourier transform [41] from the transversal accelerations, their power spectral density (PSD) and their corresponding fundamental rotating harmonic intensity are finally obtained.

**Figure 4:**
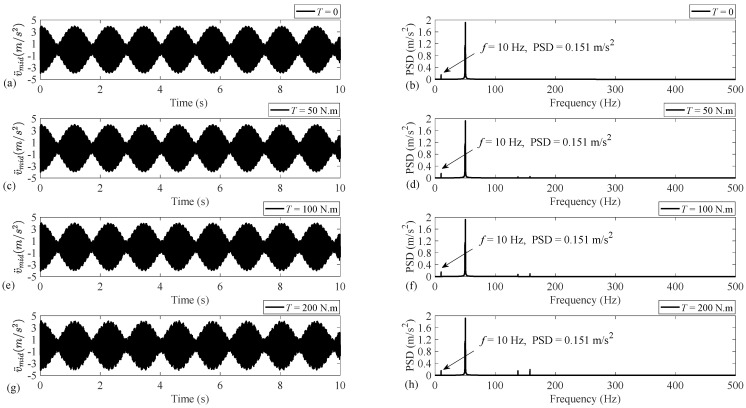
Acceleration time-history and PSD associated with the undamped disc–shaft system with properties given in Table 2: (**a**,**b**) No load, (**c**,**d**) $T_{L}=50\;N.m$, (**e**,**f**) $T_{L}=100\;N.m$, and (**g**,**h**) $T_{L}=200\;N.m$.

Regarding the universality of the present theoretical model, to obtain numerical results, a usual steel shaft system with an unbalanced disc installed at its middle, where the torsional load is also applied, is considered with the specifications listed in Table 2. As mentioned earlier, the unbalance fault is reflected on the fundamental intensity of the transversal acceleration along the *y* axis. Therefore, Figure 4 illustrates the acceleration time-history and PSD for the horizontal axis *y*. The rotational speed is set to f=10 Hz, and the results are provided for an undamped system under four different cases of $T_{L}=0,\;50,\;100,\;and\;200\;N.m$. The accelerations are obtained with the sampling frequency of 1 kHz, and the length of the time-history is set to 10 s, representing the frequency content with the frequency resolution of 0.1 Hz.

As Figure 4 demonstrates, applying the torsional load increases the magnitudes corresponding to the existing two components at frequencies 49.7 and 50.5 Hz and causes the two components at frequencies 137.9 and 157.1 Hz to appear and increase. However, the torsional load has no influence on the intensity of the fundamental harmonic at f=10 Hz as the unbalance fault indicator. So, it can be concluded that, regardless of the level of the applied torsional load, the severity of the unbalance fault can be obtained via monitoring the intensity of the vibration signal fundamental harmonic because this intensity is independent of the torsional load.

**Figure 5:**
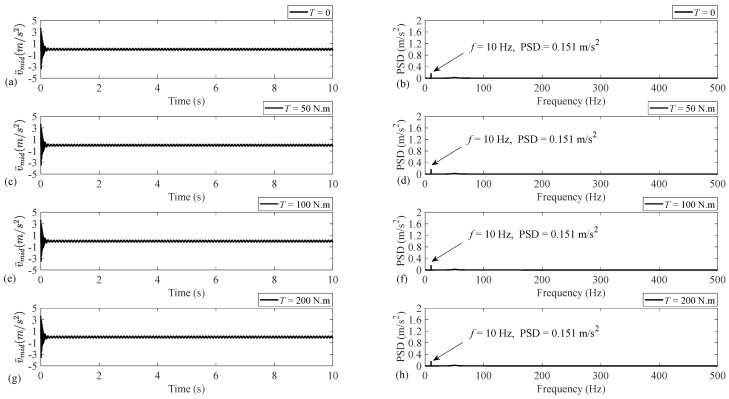
Acceleration time-history and PSD associated with the present damped disc–shaft system with properties given in table, and ${\bar{\xi }}_{y}={\bar{\xi }}_{z}={\bar{\xi }}_{t}=\bar{\xi }=0.05$: (**a**,**b**) No load, (**c**,**d**) $T_{L}=50\;N.m$, (**e**,**f**) $T_{L}=100\;N.m$, and (**g**,**h**) $T_{L}=200\;N.m$.

In view of the fact that damping is unavoidable in practical situations, taking into account the influence of damping, Figure 5 provides the acceleration time-history and PSD for the present system. The damping coefficients ${\bar{\xi }}_{y},{\bar{\xi }}_{z},\;and\;{\bar{\xi }}_{t}$, where ${\bar{\xi }}_{i}=\frac{C_{i}}{2\sqrt{M_{i}K_{i}}},\left(i\equiv y,z,t\right)$ [33], are assumed to be the same and set to $\bar{\xi }=0.05$. As Figure 5 demonstrates, except for the fundamental harmonic, all the frequency components associated with the undamped case almost disappear. This is because they are related to the transient solution, which does not play an important role in the dynamic of the system [42]. The intensities of the fundamental harmonic in the damped case are also independent of the level of the torsional load. So, the previous conclusion related to the independency of the fundamental intensity from the level of the torsional load is also verified for the damped cases.

To provide a comprehensive investigation for the influence of the torsional load on the intensity of the fundamental harmonic, Table 3 illustrates the variation in this intensity versus the torsional load for different damping ratios of $\bar{\xi }=0,0.05,0.1,$0.2, and 0.4. As this table illustrates, it can be concluded that the intensity of the vibration fundamental harmonic is independent of the level of the applied torsional load. So, since the rotational speed is constant, its novel speed-invariant diagnostic feature [23] is also load-independent.

**Figure 6:**
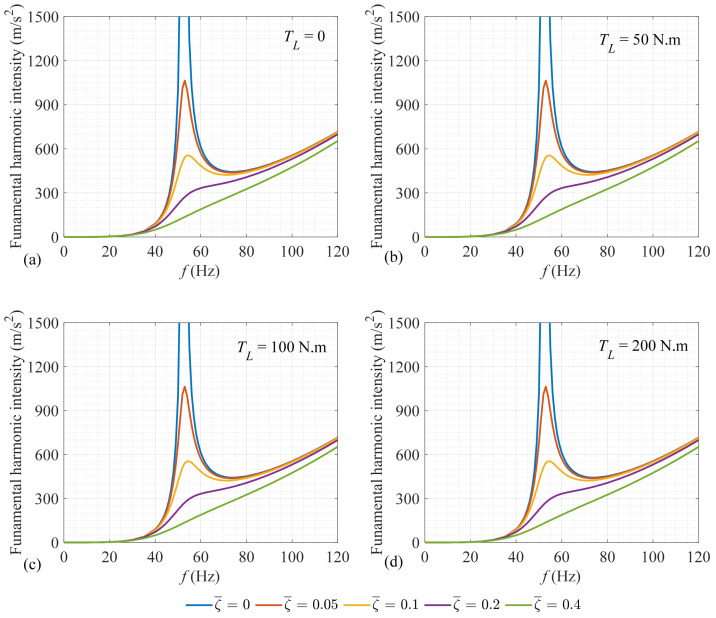
The intensity of the acceleration fundamental harmonic versus the rotational speed at different levels of damping and torsional load: (**a**) No load, (**b**) $T_{L}=50\;N.m$, (**c**) $T_{L}=100\;N.m$, and (**d**) $T_{L}=200\;N.m$.

From Table 3, it is seen that damping does not have a considerable effect on the vibration fundamental harmonic intensity. To investigate this issue, Figure 6 depicts the variation in the acceleration fundamental harmonic intensity versus the rotational speed at different load and damping levels. This figure verifies the previous conclusion, related to the independency of the fundamental intensity from the torsional load over a broad range of the rotational speeds. As this figure depicts, the difference between the damping ratio levels at areas far away from the resonance zone, where the unbalance diagnosis should be performed [4,23], is small. So, the reason behind the negligible influence of the damping level on the fundamental intensity in Table 3 is the smallness of the excitation to resonance frequency ratio for the results provided in this table, which equals 10/52.8=0.19.

To verify the theoretical conclusion related to the independency of the unbalance fault indicator from the level of the torsional load, the vibration data collected from the present experimental setup are processed. Regarding the lower stiffness of the system supports along the direction of the conveyor movement (i.e., *X* direction), the unbalance should be reflected on this direction [23]. So, the vibration data in this direction are only processed.

The vibration data are collected over a continuous 20 min time portion from the present gearmotor shell at each loading case of (i) no additional load, (ii) additional loads of 20 kg, (ii) 30 kg, and (iv) 50 kg to verify the theory. In view of the aim of the experimental analysis, where it is focused on justifying the load-independency of both the conventional and novel [23] unbalance fault indicators [23], and the collected data covering a wide range of loading cases, it will be sufficient for validating the presented theoretical background.

As mentioned earlier, the sampling frequency is 51,200 Hz. Since the nominal gearmotor rotational speed is 1440 rpm, and all the average rotational speed under loading conditions, which are given in Table 4, are around this value, the experimental data are down sampled by a factor of 10. Taking 50 s time window with 80% overlapping, the vibration data along the *X* direction are processed, and the conventional unbalance fault indicator (*S*) as well as the novel speed-invariant fault indicator (*S_N_*) are extracted. The total number of each diagnostic feature in each loading case is 116.

**Figure 7:**
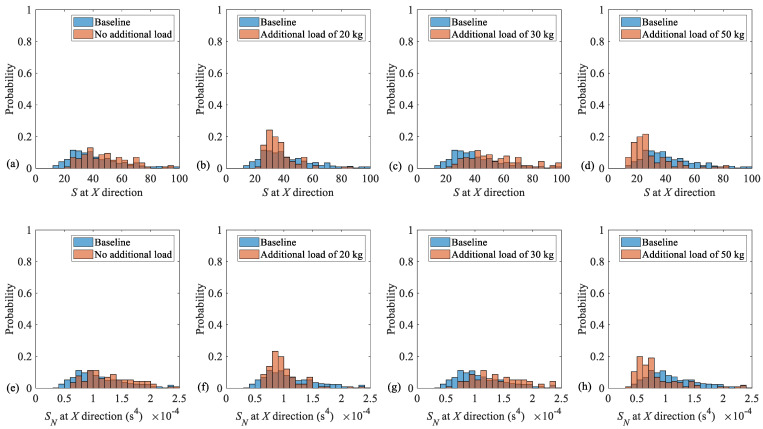
Histograms of the conventional unbalance fault indicator *S* associated with (**a**) the no additional load, (**b**) additional loads of 20 kg, (**c**) 30 kg, and (**d**) 50 kg case as well as those of the novel feature *S_N_* [23] for (**e**) the no additional load, (**f**) additional loads of 20 kg, (**g**) 30 kg, and (**h**) 50 kg case in comparison to their corresponding baselines.

Figure 7 provides histograms of both the conventional (i.e., *S*) and the novel (i.e., *S_N_*) [23] unbalance fault indicators for all four data sets and compares their probability density functions (*PDF*s) by their corresponding baselines. The baselines are obtained by combining all four data sets associated with each fault indicator. That is, the baseline *PDF*, associated with the conventional unbalance fault indicator (i.e., *S*), contains 464 features, and the novel unbalance fault indicator (i.e., *S_N_*) [23] also contains 464 features. The probabilities, corresponding to the baselines and to each loading case, are colored by blue and orange, respectively. However, the overlapping areas are colored by brown as the combination blue and orange is the default setting of MATLAB, version R2024a. As it is seen, there is no considerable separation between the four sets of data corresponding to the unbalance fault indicators associated with each loading case, in terms of neither the conventional (i.e., *S*) nor novel (i.e., *S_N_*) [23] unbalance diagnostic features, given by Equations (33) and (34), respectively.

To quantify the separation between the probabilities, Table 5 provides the values of the Fisher criterion (*FC*) corresponding to each loading case when they are comparing with the baseline. The *FC* is applied as [27]

(35) $$FC=\frac{{\left(m_{1}-m_{2}\right)}^{2}}{\mu_{1}^{2}+\mu_{2}^{2}},$$

in which $m_{1}$ and $\mu_{1}$ denote the mean value and the standard deviation of the baseline *PDF*. In addition, $m_{2}$ and $\mu_{2}$ are the mean value and the standard deviation corresponding to each loading case.

As Table 5 illustrates, there is no separation between the three sets of no additional load, additional loads of 20 kg, and 30 kg cases and their corresponding baselines either based on the conventional unbalance fault indicator or the novel speed-invariant feature [23]. However, adopting the speed-invariant diagnostic feature [23] benefits from a slightly lower separation in comparison to the conventional fault indicator. Because, as it is seen from Table 4, the application of the load has almost no effect on the average rotational speed for the three sets of no additional load, additional loads of 20 kg, and 30 kg cases. However, it slightly reduces the rotational speed in the case of 50 kg additional load.

Given the main idea of the Fisher criterion, which illustrates how far two fully separated histograms are located from each other, to have a better understanding about two overlapping histograms, it is required to investigate their separation probability (*SP*) as well [28].

To evaluate the *SP* of two histograms, their corresponding *PDF*s are first estimated under the assumption of the normal distribution for the features. Assuming normal distribution for a diagnostic feature *F*, its corresponding *PDF* is given by [28]

(36) $$PDF\left(F\right)=\frac{1}{\mu \sqrt{2\pi }}\;e^{\frac{-{\left(F-m\right)}^{2}}{2\mu^{2}}}.$$

in which m and μ denote the average and standard deviation of the feature *F*, respectively.

**Figure 8:**
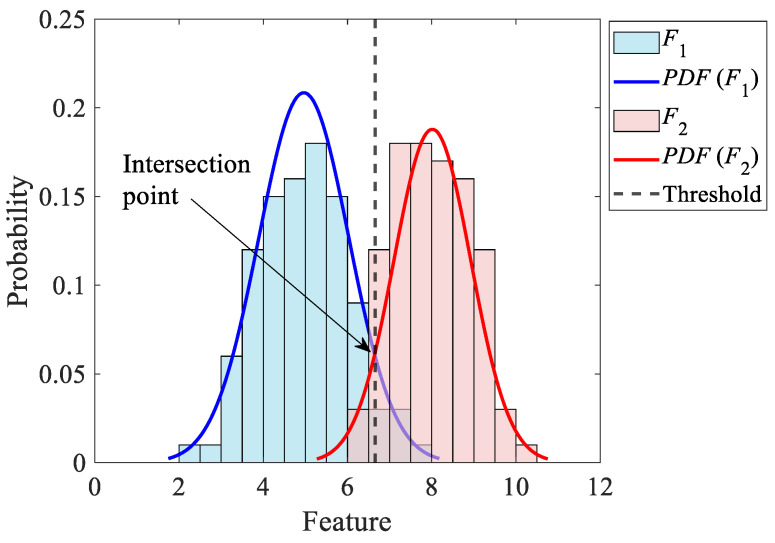
The two fitted normal *PDF*s and the intersection point.

Figure 8 demonstrates the histograms as well as the fitted *PDF*s associated with two overlapping arbitrary features. This figure also illustrates the *PDF* intersection point as the threshold. According to Figure 8, the estimates of *SP* between the two histograms are given by [28]

(37) $$SP=\frac{F_{1,\;c}+F_{2,\;c}}{F_{1,\;t}+F_{2,t}}\times 100\%,$$

in which $F_{1,c}$ is the number of features 1, whose values are less than the threshold. $F_{2,c}$ is also the number of features 2, whose values are larger than the threshold. $F_{1,t}$ and $F_{2,t}$ denote the total number of features 1 and 2, respectively. As is seen from Equation (37), the closer the probability is to 50%, the more similar the distributions of the two features are.

Table 6 represents SP estimates between all the loading cases and their corresponding baselines in terms of the conventional unbalance diagnosis feature *S*, and the novel speed-invariant unbalance indicator *S_N_* [23]. As this table demonstrates, all estimates of *SP*s are very close and very low for the conventional and novel features. This means that for all loading cases, the probability density functions are almost the same; so, the torsional loading has no considerable influence on the unbalance fault indicators. However, since despite the other cases, applying the torsional load slightly reduces the rotational speed in the case with an additional load of 50 kg, the novel speed-invariant feature *S_N_* [23] benefits from a lower *SP* in comparison to the conventional unbalance fault indicator in this case. Therefore, it is recommended to employ a speed-invariant unbalance fault indicator for unbalance diagnosis in multiple constant load conditions.

In view of the investigations mentioned above, it is experimentally seen that the torsional loading has no effect on the vibration-based unbalance fault indicators, neither on the conventional nor on the proposed [23] diagnostic features. In other words, the experimental analysis indicates that the unbalance fault indicators associated with different loading cases are similar, as there is no considerable separation between their *PDF*s. This agrees with those theoretically observed via the universal mathematical model developed earlier for unbalanced induction motors. So, regardless of the level of loading applied on an induction motor, one can monitor its unbalance condition via processing its corresponding vibration fault indicators.

## 5. Conclusions

This study proposes a novel universal analytical continuous model to investigate the coupled flexural–torsional dynamics of an unbalanced disc–shaft system as a representative of an unbalanced induction motor. Adopting the proposed model, the influence of the torsional loading on the two unbalance fault indicators is investigated for the first time wideranging.

The solutions are obtained by the combination of the Galerkin and Runge–Kutta methods. The influence of the applied torsional load on the magnitude of the transversal acceleration is studied. Taking five different cases of damping $\bar{\xi }=0$, 0.05, 0.1, 0.2, and 0.4 and simulating the variation in the fundamental harmonic intensity of the transversal acceleration over a broad range of the rotational speeds under four different loadings of $T_{L}=0$, 50 N.m, 100 N.m, and 200 N.m shows that applying torsional load on the system does not affect the intensity of its vibration fundamental harmonic as the conventional unbalance fault indicator, either in undamped cases or in the presence of damping.

The theory is verified by conducting comprehensive experimental trials with a gearmotor, running a belt conveyor system under different levels of loadings. Given the motor speed fluctuations, the vibration data along the *X* axis, which refers to the conveyor movement direction, are processed using the short-time chirp Fourier transform.

In view of the influence of load on reducing the rotational speed, to remove the effect of speed on the conventional unbalance fault indicator (i.e., *S*), the influence of torsional load on the novel speed-invariant diagnostic feature (i.e., *S_N_*) is also investigated.

It is experimentally observed that applying the torsional load does not affect either the intensity of the conventional or the novel unbalance fault indicators. The Fisher criteria, associated with the conventional and proposed features, are very low in the range (0.03–0.15).

Aside from the Fisher criterion, the estimates of separation probabilities between each loading case and the baseline corresponding to both the conventional and novel unbalance diagnosis features are also obtained. These probabilities are also very low, in the range (51.4–59.38%); these low probabilities show coincidences between the investigated feature probability density functions, which justify the conclusion, obtained via the Fisher criterion, and emphasize the load-independency of both unbalance fault indicators.

Based on the performed investigations, it is found that regardless of the level of applied torsional load, the vibration signal can be adopted to monitor the unbalance fault in induction motors. This study justifies, for the first time, wideranging the load-independency of the two vibration-based unbalance fault indicators. This makes an essential impact on unbalance diagnosis, and it is important for induction motors operating under multiple constant loads in a large number of industrial sectors.

## Figures and Tables

**Table 1:** Specifications of the triaxial accelerometer.

Model Name	354A05, PCB Piezotronics Inc.
Sensitivity	100 mV/g
Measurement range	±50 g
Operating frequency range	4 Hz–5 kHz
Resonance frequency	≥25 kHz
Nonlinearity	≤1%
Transverse sensitivity	≤5%
Operating temperature range	−54 °C to +93 °C

**Table 2:** Specifications of the disc–shaft system.

$L\left(m\right)$	$r\left(m\right)$	$r_{d}\left(m\right)$	$\rho \left(kg/m^{3}\right)$	$E\left(GPa\right)$	$G\left(GPa\right)$	$M_{d}\left(kg\right)$	$\Omega \left(Hz\right)$	$m_{im}\left(kg\right)$	$e_{im}\left(m\right)$
1	0.03	0.15	7870	206	79	50	10	0.5	0.12

**Table 3:** Influence of damping on the fundamental harmonic intensity (m/s^2^) under different levels of torsional load.

	$T_{L}=0$	$T_{L}=50\;N.m$	$T_{L}=100\;N.m$	$T_{L}=200\;N.m$
$\bar{\xi }=0$	0.151	0.151	0.151	0.151
$\bar{\xi }=0.05$	0.151	0.151	0.151	0.151
$\bar{\xi }=0.1$	0.151	0.151	0.151	0.151
$\bar{\xi }=0.2$	0.151	0.151	0.151	0.151
$\bar{\xi }=0.4$	0.150	0.150	0.150	0.150

**Table 4:** Average rotational speeds at different loading cases.

Loading Case	Average Rotational Speed
No additional load	24.68 Hz
Additional load of 20 kg	24.66 Hz
Additional load of 30 kg	24.63 Hz
Additional load of 50 kg	24.26 Hz

**Table 5:** The Fisher criterion between each loading case and the baseline probabilities in terms of the conventional unbalance fault indicator *S* and (b) the novel feature *S_N_* [23].

	*S*	*S_N_*
No additional load	0.03	0.03
Additional load of 20 kg	0.07	0.07
Additional load of 30 kg	0.07	0.07
Additional load of 50 kg	0.15	0.12

**Table 6:** The SPs between each loading case and the baseline in terms of the conventional unbalance fault indicator *S* and (b) the proposed feature *S_N_* [23].

	*S*	*S_N_*
No additional load	51.72%	51.4%
Additional load of 20 kg	59.27%	59.16%
Additional load of 30 kg	51.19%	51.08%
Additional load of 50 kg	59.38%	57.76%

## Data Availability

Data are unavailable due to privacy restrictions.
